# The Shifting Landscape of Debulking Surgery in Newly Diagnosed Advanced Ovarian Cancer

**DOI:** 10.3390/cancers18101502

**Published:** 2026-05-07

**Authors:** Dimitrios Tsolakidis, Dimitrios Zouzoulas

**Affiliations:** 1st Department of Obstetrics & Gynecology, Aristotle University of Thessaloniki, “Papageorgiou” Hospital, 56429 Thessaloniki, Greece

## 1. Introduction

Ovarian carcinoma is the third most common gynecological malignancy and the leading cause of death among breast and genital track cancers in women [1]. Due to its atypical common symptoms, ovarian cancer is usually diagnosed at an advanced stage, which negatively impacts patients’ prognosis [2]. Cytoreduction is still the cornerstone of advanced ovarian cancer treatment [3], and its theoretical objectives include reduction in tumor burden to decrease chemoresistance, optimization of tumor vascularization to enhance chemosensitivity, and decrease in tumor volume to shorten the G_0_ phase of the cell cycle in accordance with Gompertzian growth kinetics and to increase the proliferative fraction of residual disease (increased growth-factor effect) [4]. It was found that the most important negative prognostic factor after cytoreduction is the presence of residual disease, and specifically complete gross resection is the ultimate goal [5,6]. However, the timing of debulking surgery in relation to chemotherapy is still a point of controversy between researchers.

## 2. Timing of Debulking Surgery

Based on the sequence between cytoreduction and chemotherapy, three distinct debulking surgeries can be defined: primary debulking surgery (PDS), where cytoreduction is followed by chemotherapy; interval debulking surgery (IDS), where three cycles of neoadjuvant chemotherapy (NACT) are followed by cytoreduction with three additional cycles of adjuvant chemotherapy; and delayed interval debulking surgery (DIDS), where cytoreduction is performed after completion of all six cycles of NACT.

Historically, cytoreduction in the form of PDS for advanced ovarian cancer was described for the first time in 1943 by Meigs [7]; then, in 1968 Hudson first described the en block removal of the pelvic peritoneum for bulky pelvic disease [8], and in 1975 Griffiths first correlated residual disease diameter with survival rates [9]. Traditionally, debulking surgery with residual disease ≥ 1 cm is considered suboptimal, that with residual disease between 0 and 1 cm is characterized as optimal, and that with no residual disease is described as complete.

As PDS evolved through the years, the pelvis was a region that surgeons could debulk successfully with or without rectosigmoid resection in order to achieve complete gross resection, which was recently verified in a meta-analysis [10] and a study from a European Society of Gynecological Oncology (ESGO)-certified center for advanced ovarian cancer surgery [11]. However, it is known that advanced ovarian cancer usually presents with peritoneal tumor dissemination in the entire abdominal cavity, with high tumor volume in areas where gynecological oncologists are not trained to operate. A study from the early 2000s [12], investigating the limits of 640 experienced gynecological oncologist during cytoreduction, showed that unresected disease in the upper abdomen was the main reason for suboptimal debulking surgery. In a later publication from the Memorial Sloan Kettering Cancer Center [13], the authors stated that surgical ability improved over the years, leading to improved progression-free survival (PFS) and overall survival (OS). These results were further reinforced by an exploratory analysis of three randomized trials carried out by the Arbeitsgemeinschaft Gynaekologische Onkologie Studiengruppe Ovarialkarzinom (AGO-OVAR) [14], which concluded that the goal of PDS should be no residual disease, because complete gross resection showed a statistical significant improved PFS and OS benefit across all International Federation of Gynecology and Obstetrics (FIGO) stages. Furthermore, a major change in the surgery plan during PDS occurred in 2019 with the publication of the LION trial [15]. Systematic pelvic and paraaortic lymphadenectomy was abandoned in advanced ovarian cancer patients with clinically negative nodes, because the trial showed no benefit in PFS (26 months in both groups) or OS (no lymphadenectomy: 69 months vs. lymphadenectomy: 66 months). Nevertheless, patients who underwent lymphadenectomy for enlarged lymph nodes during PDS had longer surgery duration and higher rate of postoperative complications and 60-day mortality.

After 2000, NACT followed by IDS gained wider clinical approval as an alternative to PDS in select patients with advanced ovarian cancer. In 2006, a meta-analysis from the USA [16] showed that NACT followed by IDS is inferior compared to PDS, with a decrease in median OS of 4.1 months for each additional NACT cycle. On the other hand, a meta-analysis from Korea in 2009 [17] concluded that NACT helped to increase the rate of optimal debulking surgery. In the context of this contradictory evidence, three non-inferiority randomized control trials (RCTs) comparing NACT + IDS versus PDS were performed.

In 2010, the EORTC 55971 trial [18] included 670 ovarian cancer patients with FIGO stage IIIC and IV disease and found no statistical difference between the two groups in PFS (PDS: 12 months vs. IDS: 12 months) and in OS (PDS: 29 months vs. IDS: 30 months). These results showed that NACT + IDS is not inferior to PDS, while major postoperative complications were lower in the NACT + IDS group. In 2015, the CHORUS trial [19] enrolled 550 patients and presented similar results to EORTC 55971. Survival rates, PFS (PDS: 10.7 months vs. IDS: 12 months), and OS (PDS: 22.6 months vs. IDS: 24.1 months), were comparable between the two groups, and 30-day mortality rate was higher in PDS (PDS: 6% vs. IDS: <1%). However, both trials received criticism about the rate of optimal debulking surgery and surgery duration in the PDS arm, which were low, suggesting suboptimal surgical effort/experience in many centers. In addition, patient enrollment per center per year was very low, suggesting a possible selection bias towards the inclusion of unresectable and frailer patients, while “good” PDS candidates could be treated off-trial. The third randomized trial, SCORPION [20], was published in 2020 and included 171 advanced ovarian cancer patients with high tumor load (Fagotti score ≥ 8 and ≤12). In accordance with the previous studies, the authors concluded that there was no difference in PFS (PDS: 15 months vs. IDS: 14 months) or OS (PDS: 41 months vs. IDS: 43 months) between the two groups, while PDS had lower complete resection rates (PDS: 47.6% vs. IDS: 77%) and higher major postoperative complications (PDS: 25.9% vs. IDS: 7.6%), both of which were statistically significant.

In order to triage patients between PDS and IDS, three main questions should be answered regarding the insufficiency of the surgeon/center, the non-resectability of the tumor, and inoperability due to patient frailty. Surgical competence for advanced ovarian cancer surgery has been determined by ESGO with well-defined quality indicators for surgeons and centers [21]. The resectability of the disease has been subjectively based on the quality of imaging and each radiologist’s experience. However, with the implementation of diagnostic laparoscopy in the treatment algorithm [2], the extent of the disease could be more precisely evaluated. A systematic review including 1563 patients concluded that diagnostic laparoscopy could identify all unresectable disease [22], and a multicenter RCT from the Netherlands showed that this procedure reduces the number of futile laparotomies [23]. The need for a standardized and reproducible laparoscopic scoring model for disease assessment in the peritoneal cavity of suspected advanced ovarian cancer patients has led to the creation of the Fagotti score [24]. During diagnostic laparoscopy, seven areas (omental cake, peritoneal carcinomatosis, diaphragmatic carcinomatosis, mesenteric retraction, stomach infiltration, bowel infiltration, and liver/spleen metastasis) are evaluated, and a score of zero or two is given for each one, while small bowel miliary carcinomatosis is an absolute criterion of unresectability and primary debulking surgery deferral. If the sum is eight or above (≥8), NACT followed by IDS is proposed, with a diagnostic accuracy of 75% and a positive predictive value (PPV) of 100% (no risk of leaving patients unexplored that could be optimally debulked). Patient frailty has been comprehensively addressed by the Enhanced Recovery After Surgery (ERAS) society with pre-, intra-, and postoperative care recommendations for gynecological oncology patients [25,26], and by ESGO with perioperative guidelines for advanced ovarian cancer patients undergoing debulking surgery [27].

In the light of the above, the multicenter randomized trial TRUST was launched in order to settle the long-lasting debate between PDS and NACT + IDS. The main criterion for participation in the trial was the quality of surgery, as each center underwent onsite evaluation of cytoreductive surgery, while complete gross resection of 50% or more and at least 36 cytoreductions per year were prerequisites. In total, 688 patients were randomized to either PDS or NACT + IDS, with OS as the primary endpoint. In 2025, the results of the TRUST trial [28] showed that both groups were equal in terms of baseline characteristics and surgical effort, while the complete gross resection rate was higher in the NACT + IDS group (PDS: 68% vs. NACT + IDS: 79%). Concerning survival rates, the study failed to show a statistically significant difference in OS (PDS: 54.3 months vs. IDS: 48.3 months; *p*-value = 0.24), even though PDS presented with a numerically higher OS in the whole population, FIGO Stage III, and complete gross resection after subgroup analyses. On the other hand, PDS showed a statistically significantly improved PFS (PDS: 22.1 months vs. IDS: 19.7 months), which was maintained across subgroup analyses for FIGO Stage III and complete gross resection. Furthermore, PDS had a higher rate of postoperative complications (PDS: 18% vs. NACT + IDS: 12%), but no difference in the overall quality of life of the patients was observed between the two groups at any timepoint during the trial. Also in 2025, the SUROVA study was published [29]. This was an international, retrospective, multicenter real-world study, which included 3286 patients with advanced ovarian cancer. Its results complemented those of the TRUST trial and showed that postoperative complications were associated with significantly worse survival, especially for PDS, while patients who were offered primary surgery with complete gross resection and no postoperative complications had the best outcomes.

In recent years, DIDS has emerged as an alternative to IDS for primary inoperable patients with poor initial response to three cycles of NACT. The exact number of NACT cycles has not yet been established; however, in most studies, three to four cycles of chemotherapy are proposed prior to IDS. In contrast, real-world data indicate that the majority of patients are usually offered five or more NACT cycles before cytoreduction. Based on this question, the CHRONO trial [30], which randomized patients to three or six cycles of NACT before debulking surgery, was established. While we await its results, the combined data of two retrospective studies have clarified the role of DIDS in the treatment algorithm of advanced ovarian cancer. GO SOAR2 [31] was an international, multicenter, retrospective cohort study including 2498 patients; it showed no difference concerning PFS (5-year PFS, IDS: 26.5% vs. DIDS: 31.6%), but a significantly improved OS (5-year OS, IDS: 48.5% vs. DIDS: 38.2%) was observed between the two groups. These results were reinforced by the data (PFS, IDS: 17 months vs. DIDS: 18 months/OS, IDS: 52 months vs. DIDS: 36 months) of an ESGO-certified center for advanced ovarian cancer surgery [32], establishing DIDS as a safe alternative to IDS for a specific group of patients where complete gross resection was not feasible with PDS or IDS. Furthermore, a biomarker that can predict which patients will achieve complete gross resection after NACT + IDS has been investigated. The CA-125 ELIMination rate constant K (KELIM) score has evolved as an independent prognostic predictor for advanced ovarian cancer patients in the interval setting, based on the results of several trials [33,34].

Last but not least, the adaption of hyperthermic intraperitoneal chemotherapy (HIPEC) in the treatment algorithm of advanced ovarian cancer has given rise to a debate about its role and possible limitations. At this point, there are no available robust data to support the use of HIPEC after PDS, and researchers are awaiting the results of the OVIHIPEC-2 trial [35] to assess the safety and oncological outcomes of HIPEC in the primary setting. On the other hand, in the interval setting, the role of HIPEC after NACT + IDS has been established after the OVIHIPEC-1 trial [36] in 2018. This study showed that HIPEC after IDS was associated with a significant improvement in PFS (IDS + HIPEC: 14.3 months vs. IDS: 10.7 months) and OS (IDS + HIPEC: 44.9 months vs. IDS: 33.3 months), compared to IDS alone. However, questions about treatment-related adverse events were raised, and criticism was directed towards an increased rate of postoperative complications in the HIPEC group. A recent study from an ESGO- and European School of Peritoneal Surface Oncology (ESPSO)-certified center [37], which included patients who underwent either PDS or IDS, concluded that HIPEC after cytoreduction was associated with improved OS, but also with a higher rate of postoperative complications.

## 3. Conclusions

The timing of debulking surgery in advanced ovarian cancer patients has changed in recent decades. However, the goal is always to achieve complete gross resection as early as possible in the treatment algorithm. Thus, it is obvious that the key lies in the correct selection of patients who will be the best candidates for PDS, IDS, or DIDS, in order to optimize survival outcomes.

## Data Availability

No new data were created.

## Abbreviations

The following abbreviations are used in this manuscript:

PDS	Primary Debulking Surgery
IDS	Interval Debulking Surgery
DIDS	Delayed Interval Debulking Surgery
NACT	Neoadjuvant Chemotherapy
ESGO	European Society of Gynecological Oncology
ESPSO	European School of Peritoneal Surface Oncology
PFS	Progression-free Survival
OS	Overall Survival
AGO-OVAR	Arbeitsgemeinschaft Gynaekologische Onkologie Studiengruppe Ovarialkarzinom
FIGO	International Federation of Gynecology and Obstetrics
RCTs	Randomized Control Trials
PPV	Positive Predictive Value
KELIM	CA-125 ELIMination rate constant K
HIPEC	Hyperthermic Intraperitoneal Chemotherapy
